# Threshold Equalizing Noise Test Reveals Suprathreshold Loss of Hearing Function, Even in the “Normal” Audiogram Range

**DOI:** 10.1097/AUD.0000000000001175

**Published:** 2022-03-11

**Authors:** Michael A. Stone, Emanuele Perugia, Warren Bakay, Melanie Lough, Helen Whiston, Christopher J. Plack

**Affiliations:** 1 Manchester Centre for Audiology and Deafness, School of Health Sciences, University of Manchester, Manchester, United Kingdom; 2 Manchester University Hospitals NHS Foundation Trust, Manchester, United Kingdom; 3 Institut d’Audition, Institut Pasteur, Paris, France; 4 Department of Psychology, Lancaster University, Lancaster, United Kingdom.

**Keywords:** NESI, Noise-induced hearing loss, Subclinical hearing loss, TEN test

## Abstract

**Objectives:**

The threshold equalizing noise (TEN(HL)) is a clinically administered test to detect cochlear “dead regions” (i.e., regions of loss of inner hair cell [IHC] connectivity), using a “pass/fail” criterion based on the degree of elevation of a masked threshold in a tone-detection task. With sensorineural hearing loss, some elevation of the masked threshold is commonly observed but usually insufficient to create a “fail” diagnosis. The experiment reported here investigated whether the gray area between pass and fail contained information that correlated with factors such as age or cumulative high-level noise exposure (>100 dBA sound pressure levels), possibly indicative of damage to cochlear structures other than the more commonly implicated outer hair cells.

**Design:**

One hundred and twelve participants (71 female) who underwent audiometric screening for a sensorineural hearing loss, classified as either normal or mild, were recruited. Their age range was 32 to 74 years. They were administered the TEN test at four frequencies, 0.75, 1, 3, and 4 kHz, and at two sensation levels, 12 and 24 dB above their pure-tone absolute threshold at each frequency. The test frequencies were chosen to lie either distinctly away from, or within, the 2 to 6 kHz region where noise-induced hearing loss is first clinically observed as a notch in the audiogram. Cumulative noise exposure was assessed by the Noise Exposure Structured Interview (NESI). Elements of the NESI also permitted participant stratification by music experience.

**Results:**

Across all frequencies and testing levels, a strong positive correlation was observed between elevation of TEN threshold and absolute threshold. These correlations were little-changed even after noise exposure and music experience were factored out. The correlations were observed even within the range of “normal” hearing (absolute thresholds ≤15 dB HL).

**Conclusions:**

Using a clinical test, sensorineural hearing deficits were observable even within the range of clinically “normal” hearing. Results from the TEN test residing between “pass” and “fail” are dominated by processes not related to IHCs. The TEN test for IHC-related function should therefore only be considered for its originally designed function, to generate a binary decision, either pass or fail.

## INTRODUCTION

Degradation of the mammalian auditory system has been shown to be caused by a variety of factors such as age, genetics, oto-toxic pharmaceuticals, and noise exposure (Schmiedt 2010; Op de Beeck et al. 2011; Böttger & Schacht 2013). Elevation of the audiogram, a measure of the minimum detectable level of pure tone when presented in silence, is routinely used to quantify the degree of hearing loss. It has long been understood to be insufficient in predicting performance on tasks requiring suprathreshold discrimination (Hirsh et al. 1950). Although it can be used as a predictor of the ability in the more everyday suprathreshold task of decoding speech-in-noise (Harris 1965; Glasberg & Moore 1990a; Smoorenburg 1992), its prediction accuracy can be less than that obtainable by measure of other suprathreshold tasks (Glasberg & Moore 1990a) or confounded by coexisting retrocochlear pathologies, such as auditory neuropathy (Starr et al. 1996). The insufficiency of the audiogram to predict suprathreshold performance is not surprising since, even for a similar degree of loss, participants show a wide range of performance on suprathreshold tasks (Alvord 1983; Glasberg & Moore 1990a; Strelcyk & Dau 2009; Kortlang et al. 2016).

Hearing deficits can be observed even before the audiogram shows a loss of sensitivity beyond the range of “normal.” Clinically, this can take the form of measured difficulties with speech perception in noise (prevalence of approximately 8%, Stephens et al. 2003), or tinnitus (similar prevalence of 8%, Barnea et al. 1990). Although early animal experimentation showed that noise exposure caused physical damage to the structures of the cochlea (Spoendlin 1971), this could occur with no change in the audiogram, even though there may have been observable physical damage (Henderson et al. 1974). Noise-induced damage has been observed at multiple cochlear sites such as the stria vascularis, the inner and outer hair cells (IHCs/OHCs), and their associated substructures such as stereocilia, in animals (Spoendlin 1971; Liberman & Dodds 1984; Liberman & Kiang 1984), and spiral ganglion cells in humans (Otte et al. 1978). Loss of hearing function, independent of observable physical damage (where observation is permissible), and where there is no apparent change in the audiogram, may be classified as a “subclinical” loss. A more popular term, “hidden hearing loss” (Schaette & McAlpine 2011), has acquired multiple definitions across reports (Pienkowski 2017; Bramhall et al. 2019) so that, for this article, we use the more precise label “subclinical,” meaning a loss that is not detectable by current clinical processes, that is, classified as “normal” hearing (audiometric thresholds in the range ≤ 20 dB HL).

There is a considerable interest in the development of measures applicable to humans to identify the presence of, and tools to monitor the progression of, subclinical losses, as well as a differential diagnosis to identify possible site(s) of lesion. Such identification (by employing measures such as oto-acoustic emissions [OAEs], Attias et al. 1998; Hall & Lutman 1999; Sliwinska-Kowalska & Kotylo 2001; Lucertini et al. 2002; psychophysical tasks, Stone et al. 2008; Ridley et al. 2018; electrophysiology, Bharadwaj et al. 2015; Skoe & Tufts 2018; extended high-frequency audiometry, LePrell et al. 2013; Sulaiman et al. 2013) could be used as an early-warning system in groups whose lifestyle, or genetic predisposition, places them at risk of an avoidable accelerated hearing damage. Although many of the studies cited primarily focus on monitoring the effects of noise-induced loss, the tools are readily transferable to investigate other agents of damage, such as the monitoring of the effects of oto-toxic pharmaceuticals, whose action may differentially affect subcomponents of the cochlea (e.g., Konrad-Martin et al. 2010). There is a growing consensus that no single test will produce a high degree of differential diagnosis and therefore a battery of tests will be required (Lopez-Poveda & Johannesen 2012; Bharadwaj et al. 2015; Ridley et al. 2018; Verhulst et al. 2018).

The experiment reported here was part of larger experiment, again using a psychophysical test battery approach, that followed up on the findings of Stone et al. (2008) and Stone & Moore (2014). These reports identified putative IHC-related impairments due to high-level noise exposures from nightclubs and amplified music concerts (“gigs”), typically with sound pressure levels (SPLs) exceeding 100 dBA. The hypothesis was that, in line with the demonstration of a “Critical Intensity” (Ward et al. 1981), more precisely observed in animals, exposures above a certain level would manifest as a different pattern of hair cell damage in humans, when compared with the pattern observed for exposures below the critical intensity. Harding and Bohne (2004, p. 2219) suggested that the definition of critical level “…should not be limited to the threshold for mechanical damage.” and “…should be expanded to include the level at which substantial secondary hair-cell loss occurs post-exposure.” Stone et al. demonstrated the possible effect of a critical intensity in humans by the use of low sensation level (SL) signals (typically ≤ 20 dB SL). The choice of low-SL testing was made so that neural transduction occurred close to the place of the test frequency and therefore entrained relatively few suprathreshold neurons as well as operating on a more linear portion of the basilar membrane vibration dependence on level (reducing a possible confound of the influence of cochlear compression). Additionally, transduction of low-SL signals introduces little or no extra broadening of the auditory filter, limiting spread of cochlear excitation, thereby providing a second approach to limiting the number of entrained neurons. It was hypothesized that limiting the region of transduction would be more likely to show up even patchy cochlear damage. A separate study (Vinay & Moore 2010), also using low-SL signals, has reported results also differing according to degree of noise exposure, but in groups identified by their relative use of personal music players (PMPs). PMPs are rarely used at levels above 85 dBA (~20%, Twardella et al. 2016), except in high levels of external background noise where, even there, levels very rarely exceed 100 dBA (Worthington et al. 2009; Keith et al. 2011; Shimokura & Soeta 2012). These low-SL studies all used small subject groups (N typically < 40), so may have been underpowered. There was therefore a need to expand the range of tests employed, as well as increase the number of participants.

Studies using low-SL presentations employ a different reasoning from studies investigating cochlear synaptopathy, an effect first demonstrated in rodents where cochlear damage, specifically loss of IHC synapses, was observed with no change in absolute threshold (Kujawa & Liberman 2009). Noise-induced synaptopathy may primarily affect neurons with low spontaneous rates (rodents, Furman et al. 2013), which led to the prediction that such effects would only be observable at high-SL testing. Many of the test batteries listed earlier (Lopez-Poveda & Johannesen 2012; Bharadwaj et al. 2015; Ridley et al. 2018; Verhulst et al. 2018) were explicitly looking for synaptopathy in humans and therefore have used high-SL presentations. Deficits at low SLs cannot easily be attributed to damage to fibers with low spontaneous rates (due to their relative lack of abundance compared with fibers with high spontaneous rate), implying the possibility of a different mechanism of damage from that used to justify high-SL testing.

A battery of tests used to perform a clinical site-of-lesion diagnosis costs clinical time, and has yet to be implemented in a cohesive structure. Some of the tools identified above (such as OAEs and electrophysiology) are available clinically. One clinical tool that offers a differential diagnosis of the likely cause of dysfunction is the threshold equalizing noise (TEN) test (Moore et al. 2004). In the TEN test, a participant is required to detect tones presented in a uniformly masking wide-bandwidth noise. Given a priori assumptions about the variation with frequency of both filter shape and detection efficiency, the scale of the threshold measure can be chosen to be equal in either dB SPL or dB HL. The TEN test used here, being from a clinical test, produces nearly equal masked thresholds on the dB HL scale. The noise intensity, usually specified in dB HL/ERBn, the intensity within one auditory filter of “normal” width (Glasberg & Moore 1990b), is set at a minimum of 10 dB above absolute threshold for the tone, and the tone level adjusted until detection of the tone is achieved. When there is no cochlear dysfunction, the level of the tone should be within a few dB of the calibrated noise intensity. If the detection threshold is elevated by more than 10 dB above the level of the noise intensity, then a “dead region” is diagnosed. A “dead region” is where there is no in-place transduction of the tone from physical vibration of the basilar membrane to a neural signal, and its presence is detected by regions of the ascending neural pathway to either side of the dead region, where there is surviving transduction. Although the terminology used is of a “cochlear dead region,” the lack of transduction indicates a loss of neural pathway between vibration and cortical detection, and therefore incorporates multiple structures on the ascending auditory pathway. The TEN test does not necessarily indicate that the IHC itself is actually the site of lesion, but it does discriminate between the IHC-pathway and OHC-related processing. As a clinical test, it is quick and easy to administer.

As originally developed, the TEN test results in a binary decision: pass or fail at each frequency tested. However, anecdotal reports observe some elevation of the detection threshold in individuals with hearing impairment. Some of this elevation was expected, as described in the original version of the TEN test (Moore et al. 2000): damage to OHCs could be expected to produce broadening of the auditory filters, integrating more noise within their passband, and making a tone harder to detect. The worst-case elevation in detection threshold as a result of the broadening was expected to be around 2 to 3 dB, but the associated filter broadening, a factor of 3.8, is normally only observed for severe degrees of hearing loss (Moore & Glasberg 2004). Apart from OHC damage, filter broadening can also occur when high replay levels of the TEN noise are used, even in participants with normal hearing (Glasberg & Moore 1990b). In practice, high testing levels affect the elevation of the TEN threshold differently across frequencies, something not seen at lower testing levels of 30 and 50 dB HL/ERBn (Hansen et al. 2017). At a presentation level of 70 dB HL/ERBn, Hansen et al. (2017) reported an elevation of around 1 dB for frequencies at or below 1 kHz, but rising to over 2 dB at 3 and 4 kHz. Therefore, any elevation of the masked TEN threshold can be expected to be due to contributions from two structures, the OHC, and the IHC neural pathway, but will eventually be dominated by the latter when a “fail” criterion is reached.

The primary hypothesis behind this report is that elevation of the TEN threshold, insufficient to be classed as a “fail,” may indicate the onset of a “sick,” rather than a “dead” region. Identification of such could provide an early warning, such as behaviour modification, well before the perceptual consequences of a dead region become apparent. In this sick region, we would expect to see the balance between the OHC and IHC contributions gradually shifting, but possibly with a dependence varying with frequency. For example, noise-induced damage in humans is typically first observed clinically as a notch in the audiogram between 3 and 6 kHz (Fowler 1929; Coles et al. 2000). If the human 3 to 6 kHz region is more susceptible to noise damage, then a search for subclinical markers of this damage would involve a comparison of elevation of the TEN thresholds within and outside of this frequency region and should show correlations with noise-exposure measures, such as the Noise Exposure Structured Interview (NESI, Guest et al. 2018). We therefore expected to see, for the same absolute threshold, an excess elevation in the masked TEN threshold (over any effect of filter broadening) that correlated with the cumulative noise exposure, but primarily in the 3 to 6 kHz region, and little effect of cumulative noise exposure in the 0.75 and 1 kHz region. A secondary hypothesis was that these elevations should be more strongly correlated with measures of noise exposure that are based on very high SPL exposures, >100 dBA, levels that are similar to or exceed the Critical Intensity observed in animals, suggestive of a shift in relative contributions between OHC- and IHC-related damage.

This study reports results from the use of the TEN test during the screening of a participant pool for the “battery” project mentioned above. In particular, the experiment had certain similarities to that reported by Ridley et al. (2018), but differed in several major ways. Ridley et al. (2018) recruited a total of 33 adult subjects, split between two groups, one with normal hearing with an age range of 23 to 48 years, and one with normal hearing up to 1 kHz, but also with elevated thresholds at 4 kHz and ranging in age between 35 and 64 years. Hence, a possible confound of age may have been present when group-wise analyses were performed. All participants completed an interview on their noise-exposure history. They performed a battery of tests involving electrophysiology, OAEs and loudness scaling at 1 and 4 kHz, and used their results to model the residual variance of the threshold elevation of the TEN test unaccounted for by the absolute thresholds at 1 and 4 kHz. Because they were investigating the possible manifestation of (primarily noise-induced) human cochlear synaptopathy, based on the findings of Furman et al. (2013), they used a very high level of 70 dB HL/ERBn in the TEN test. Even with normal hearing, this level would generate an extra broadening of the auditory filters of at least 20% over the width observed at lower presentation levels, resulting in extra integration of the TEN noise and, in an elevated detection threshold. Use of a high test level therefore introduces additional possible confounds to experimental results, this time directly related to normal, and not impaired, cochlear function.

Another test that is possible to administer clinically in a short period of time is the “Temporal Fine Structure—Adaptive Frequency” (TFS-AF) test, a test of acuity to binaurally presented temporal fine structure (Füllgrabe et al. 2017). It adaptively measures the highest frequency at which an inter-aural phase difference (IPD) between pulsed tones can be detected. Whereas the TEN test is a test based on detection and assesses a monaural connectivity of the IHCs, the TFS-AF test can be seen as a test based on discrimination, and by relying on the phase of the neural coding, it assesses the fidelity of binaural transduction by the IHCs and their ascending neural pathway. The TFS-AF test was therefore also included in the experiment to be reported since, in requiring similar IHC-pathway function in both ears, it was hypothesized as being more sensitive to IHC-pathway dysfunction. However, since the TFS-AF test result is only a single “figure of merit,” it is not as frequency-specific as the TEN test. The two tests therefore provide potentially complementary information.

Our recruitment sought older participants, because the previous reports using low-SL testing had selected younger people (group means < 35 years) with, at most, mild losses. Since it arose from a screening process, our recruitment was less targeted and less selective than that of Ridley et al. (2018), with the intention to explore a wider range of impairment and ages as well as a larger number of participants than in previous low-SL work. A further difference was employing more probe frequencies in the TEN test, four rather than just the two of Ridley et al. (2018). As well as controlling for the potential confounds of group age differences and high testing levels, we also generated a proxy measure of music experience, a factor which can influence performance in psychophysically derived suprathreshold test results (Parbery-Clark et al. 20 09; Yeend et al. 2017; Perugia et al. 2021).

## MATERIALS AND METHODS

### Participants

Participants were recruited for screening whose self-reported lifestyle of noise exposure might have caused sensorineural damage. Since the initial recruitment was on the basis of lifestyle and not reported hearing difficulties, it was expected that some would have normal or near-normal hearing. For the purposes of the experiment described here, the detection of subclinical losses, these participants were retained.

The selection criteria for passing this screening were that participants were as follows: greater than 18 years of age, fluent speakers of English since birth, in generally good health for their age, physically able to travel for testing, and available for multiple sessions, if successful.

Clinically, it was intended that they:

(a) had no underlying neurological problems or history of head trauma,
(b) had never worn hearing aids in the past (and so were previously “subclinical“),
(c) were audiometrically likely to benefit from a hearing aid, that is, they had a mild-moderate high-frequency hearing loss (NICE 2018), here more rigidly defined as a minimum of 30 dB and a maximum of 70 dB threshold elevation between 3 and 6 kHz, both ranges referenced to their better ear,
(d) had no history of major middle ear dysfunction, and an intact tympanic membrane,
(e) had a negligible conductive component to the hearing loss (≤10 dB).

For the experiment reported here, condition (c) was only enforced to set an upper limit to their hearing loss to select participants to go forward to further testing (reported in Perugia et al. 2021).

After this initial screening, participants were excluded from further consideration if they had

(f) a moderate hearing loss in the better ear, defined as a minimum of 41 dB HL, based on the average of the pure-tone air conduction (AC) hearing threshold levels from 0.5 to 6 kHz, including half octaves (BSA 2018). This excluded people who should already be wearing a hearing aid,
(g) a threshold elevation above 15 dB HL at 0.75 and/or 1 kHz, the reasoning for this will be detailed later,
(h) no episodes of noise exposure exceeding 100 dBA.

Routine audiological screening was performed bilaterally, consisting of otoscopic examination of the external auditory meatus, tympanometry, bone conduction, and AC audiometry. In addition, all participants were interviewed by the experimenter so as to complete the NESI (Guest et al. 2018). The NESI has been effective in tinnitus classification (Guest et al. 2017) and has been shown to correlate with a measure of noise-induced cochlear damage (Shehorn et al. 2020).

Of the initial 167 participants tested, a total of 51 were excluded from further consideration due to violating one or more of conditions (f), (g), and (h) earlier. These numbered 11, 24, and 18 participants, respectively.

Criterion (g), having low-frequency thresholds within the “normal” range was used as a proxy to select for participants who we expected to have had well-within-normal hearing at birth, and our observations were of the hearing status after some postnatally acquired hearing loss. In addition, measures of hearing ability at low frequencies could also act as a within-participant statistical control for any differential effects of noise exposure across frequency, such as that expected to primarily affect the 3 to 6 kHz region. The choice of 15 dB HL, rather than the more common 20 dB HL, will also be explained later.

Of the remaining 116 participants, 74 were female. The group mean age was 51.8 years with a median of 53 years, and with a range of 21 to 91 years. All participants were paid an honorarium for their attendance, as well as travel expenses. The remaining testing, described later, except for those undergoing TFS-AF, was performed unilaterally on the better ear, as defined by the AC audiometry.

The study received ethical approval from the NRES Committee North West—Greater Manchester Central (REC number 16/NW/0260).

### Method

The tones in the TEN test are usually presented continuously, as per manual audiometry. However, some pilot trials showed that participants with tinnitus performed more reliably when the probe tone was pulsed, rather than the usual continuous-presentation method. Lentz et al (2017) recommend the use of pulsed tones over warble or steady tones when tinnitus is present to obtain more accurate audiograms. The tones were therefore presented pulsed. The tones were ramped on with raised-cosine ramps with a duration of 15 ms, maintained a steady level for 225 ms, and then ramped off with a raised-cosine ramp of 15 ms duration. The inter-burst interval was 105 ms. The burst presentation rate was therefore 2.8/s. The relative level between the steady portion of the tone bursts and the noise was left unchanged from the original test. The TEN noise was left unaltered.

The TEN test was administered by replay off a CD player (Topaz CD5, Cambridge Audio, United Kingdom), routed through an audiometer (Madsen Astera, GN Otometrics A/S, Denmark), and delivered via a single earpiece of a TDH39 headphone (Telephonics, United States). The level of the target tone was adjusted in 2-dB steps and presentation controlled via manual audiometry. The AC absolute threshold (to the pulsed tones) was obtained at four frequencies, 0.75, 1, 3, and 4 kHz, and the TEN threshold measured with noise densities of 12 and 24 dB SL relative to these absolute thresholds. The TEN thresholds were transformed to calculate the elevation of the tone threshold relative to the TEN noise level. In line with Hansen et al. (2017), we refer to this as the “Signal-to-TEN Ratio” (STR), in units of dB.

The design of the TEN spectral shape (spanning 0.3 to 7 kHz, Moore et al. 2004) was influenced by the “detection efficiency” of the participant, which reflects the signal-to-noise ratio at which the tone can be detected in the noise. This efficiency can also vary according to presentation method (and other factors such as statistics of the noise). With normal hearing, this efficiency is –3 dB at 1 kHz when using a computer-tracked procedure, but it is closer to 0 dB when using manual audiometry (Moore et al. 2004, p. 482). The use here of a pulsed presentation with manual audiometry was closer to a computer-tracked procedure since the regularity of the pulsing indicates to the observer when to “look.” Hence, we expected that the range of elevations of TEN threshold that we observed would be shifted downward relative to those obtained from the regular TEN(HL) test. This lowering would also be true for the absolute thresholds obtained by pulsed tones, and has been reported, on average to be approximately 2 dB (Lentz et al. 2017). The decision in this article to use the more conservative figure of 15 dB HL as the upper bound for “normal” hearing is based on this finding (where the absolute threshold was obtained by pulsed tones).

The NESI (Guest et al. 2018) was then administered by the experimenter and entered into a spreadsheet for consistent computation of a cumulative noise exposure. The interview took between 15 and 30 minutes to complete, depending on the complexity of the history. During this interview, participants reported usage of personal listening devices (such as PMPs and phones), and identified noisy activities (such as recreational, occupational, educational, and firearm) of level *L* dB SPL, in which they had engaged over their lifetime, and their duration (number of hours per day, *H*, days per week, *D*, weeks per year, *W*, and number of years, *Y*), and hearing protection usage (if any).

The sound level of these activities (units of dBA) was estimated by the participants based on recall of the vocal effort required to hold a conversation in each activity. For instance, an activity with estimated noise of 99 dBA would require the participant to shout from 4 feet (1.2 m) to hold a conversation. The calculation procedure to estimate the cumulative exposure is detailed in Guest et al. (2018). One noise exposure unit is equivalent to one working year (2080 hours) of exposure to 90 dBA.

Finally, a subgroup of 86 participants (56 female) performed the TFS-AF test. The IPD was set to 180^o^. However, thresholds obtained from listeners with normal hearing range between 1100 and 1700 Hz (Füllgrabe et al. 2017), so the TFS-AF test only required use of the frequency region where our participants had normal or near-normal hearing, and where noise-related damage is not observed in the audiogram. Poorer performance in the TFS-AF test has been linked to both age and low-frequency hearing loss (Füllgrabe et al. 2018). It should be noted, however, that the youngest participant was 61 years in Füllgrabe et al. (2018), and so would be placed near the upper end of the age range of our participants. Stimuli were presented through ER 2 insert earphones (Etymotic Research Inc, Elk Grove, IL). The reasons why not all of the 112 participants completed this test were any of time limitations, equipment-output limitations, or a markedly asymmetric hearing loss.

### Statistical Analyses

Both correlational analyses and mixed-effects modeling were employed. Pearson correlations were used to explore contradictory claims about the relationship between NESI and Age (Smith et al. 2000; Prendergast et al. 2017b, 2019) and possible effects of NESI on TFS-AF threshold. This latter relationship could indicate a putative damage to phase coding in IHC due to noise exposure. Participants were stratified according to degree of hearing loss, noise exposure, and music experience: details of these groupings will be given later. Since these distributions were not continuous, Spearman correlation coefficients for ranked data were performed on the entire cohort, to evaluate the relationships of absolute threshold and STR as a function of frequency, hearing group, and age.

Mixed models were performed separately for the absolute thresholds and STRs. In these models, absolute threshold, and STRs were entered as dependent variables; frequencies, presentation level (12 or 24 dB SL), hearing, noise, and music group were evaluated as fixed effects.

These analyses were performed to generate evidence for the hypotheses mentioned in the Introduction by asking: (1) is a gradual shift in balance between OHC-related (e.g., filter broadening) and IHC-related deficits (observable within a “dead” region) as the STR becomes elevated demonstrable in the data, and, (2) does amount of noise exposure, as measured by NESI100 (while controlling for other factors such as absolute threshold, age (over and above the elevation of absolute threshold by presbyacusis and music experience)) dominate elevated STRs?

All statistical analyses were performed in R (version 3.6.3, R Core Team 2020) via R Markdown (Xie et al. 2018; Allaire et al. 2020; Xie et al. 2020). Data are visualized within *ggplot2* (Wickham 2016) using *Raincloud* (Allen et al. 2019). Durbin-Watson tests for multiple linear regression models were performed via *lmtest* (Zeileis & Hothorn 2002). The mixed models were fitted and evaluated using the packages *lme4* (Bates et al. 2015), *lmerTest* (Kuznetsova et al. 2017), and *performance* (Lüdecke et al. 2020). Post hoc pairwise comparisons were conducted via the estimated marginal means using *emmeans* (Lenth 2020) with Kenward-Roger approximation for degrees of freedom and Bonferroni correction for multiple comparisons.

## RESULTS

### Groupings Used in the Analyses

A final stage of exclusion was based on the statistical distribution of the final group so that there were no wild outliers when stratified by age (N = 4, three for being less than 30 years, and one much greater than 74 years).

**Fig. 1.**
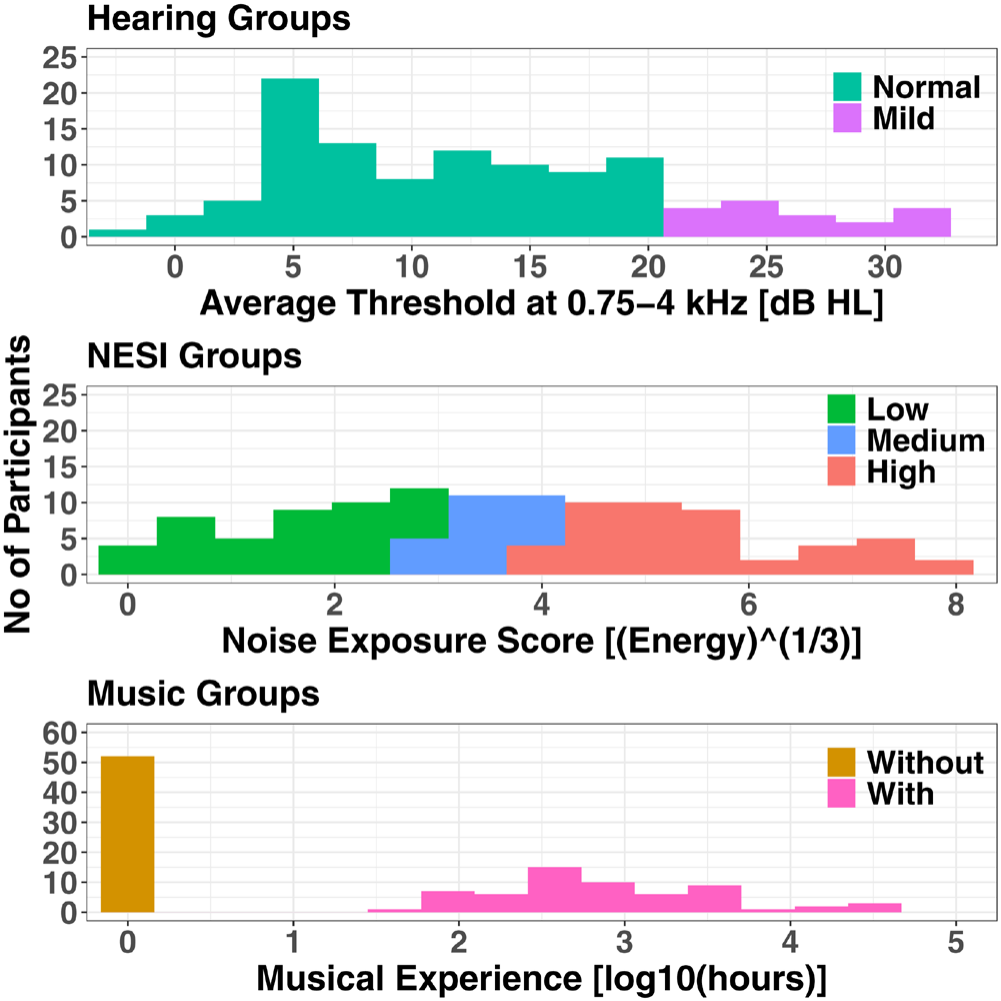
Histograms of participant measures, grouped by color into either two groups (by hearing status, top panel, or music experience, lower panel), or three groups, noise exposure (NESI score, middle panel).

Figure 1 shows the groupings generated for the analyses according to degree of hearing impairment (normal or mild), NESI (low, medium, or high), and music experience (without or with). Only the data from the low and high noise-exposure groups were examined in these models. Since the NESI relies on historical recall, poor recall would reduce the precision of the measure and blur any boundaries between groupings. The separation may increase the likelihood of observing the effects of noise exposure as a difference between groups if there are floor or ceiling effects (see Prendergast et al. 2017a,b).

### Degree of Hearing Impairment

The degree of hearing impairment of each participant was calculated as the mean AC threshold obtained by manual audiometry, averaged over the same test frequencies as used in the TEN test, 0.75, 1, 3, and 4 kHz. Participants with a mean exceeding 20 dB HL (N = 18, 10 female) were classified as having “mild” hearing impairment, the remainder had “normal” hearing. Their distribution is shown in Figure 1, top panel. The “normal” group comprised 94 participants (61 female) with a mean PTA of 10.0 dB HL, and age range 32 to 74 years (mean of 51.1). It should be noted that the use of a 20 dB HL boundary between “normal” and “mild” hearing loss has been argued as being too lenient, given the distribution of hearing thresholds in young normal-hearing listeners (Pienkowski 2017).

### Cumulative Noise Exposure

The noise exposure interviews of Stone et al. (2008) and Stone & Moore (2014) were only focused on quantifying exposures to recreational noise where the level was estimated to exceed 100 dBA. To parallel the hypotheses of these earlier studies, the NESI cumulative exposure measure was computed in two ways:

(1) conventionally, as cumulative exposure for all exposures where the sound level was estimated to exceed 80 dBA, which corresponds to all exposures recorded by the NESI, and is likely to capture exposures from PMPs. We refer to this measure as “NESI80.”
(2) cumulative exposure for exposures where the sound level was estimated to exceed 100 dBA, more in line with the exposures recorded by Stone and colleagues. This calculation of the NESI will be referred to in figures and Tables as “NESI100.”

In the statistical analyses to be presented, the pattern of the results when modeling with the NESI8 0 scores was very similar to that for the NESI100 scores, and so NESI80 scores will not be considered further except to address the secondary hypothesis from the Introduction that the pattern of results should vary depending on whether NESI80 or NESI100 was used as the noise-exposure metric. However, to capture music experience, the NESI80 data set was required.

The cumulative units of exposure were cube-root transformed to obtain a distribution approximately Gaussian. This scaling was also used in Stone & Moore (2014). All data from participants forming outliers in this distribution were discarded following the exclusion criteria detailed earlier.

The distribution of NESI100 scores is shown in Figure 1, middle panel. For the purposes of later statistical analysis, the participants have been split into three groups, with the boundaries chosen so that there is a good separation in the NESI scores between participants at the edge of each group, and that the minimum group size exceeded 20.

The low-exposed group comprised 43 participants (28 female), with a mean PTA of 11.3 dB HL, and age range 38 to 74 years (mean of 54.9 years). The medium-exposed group comprised 23 participants (10 female), with a mean PTA of 16.3 dB HL, and age range 32 to 69 years (mean of 51.8 years). The high-exposed group comprised 46 participants (33 female), with a mean PTA of 11.9 dB HL, and age range 33 to 68 years (mean of 49.9 years).

### Music Experience

The NESI data were further processed to produce the cumulative number of hours spent in practising a musical instrument, including in choirs. Since these data were only originally captured for exposures exceeding 80 dBA then some musicianship may have been under-quantified, if the preferred instrument was very quiet, for example, lute or acoustic guitar. Our measure therefore should be regarded as a proxy measure, hence its stratification into categories for the purpose of analysis of the data. Fifty-two participants (30 female) had no music experience, 60 (41 female) had some experience or were expert musicians.

The distribution of hours of music experience is shown in Figure 1, lowest panel, split into the two categories detailed earlier. The without-music group comprised had a mean PTA of 12.3 dB HL, and age range 34 to 74 years (mean of 53.0 years). The “with-music” group had a mean PTA of 12.8 dB HL, and age range 32 to 70 years (mean of 51.5 years).

### Distribution of Noise Exposure as a Function of Age

**Fig. 2.**
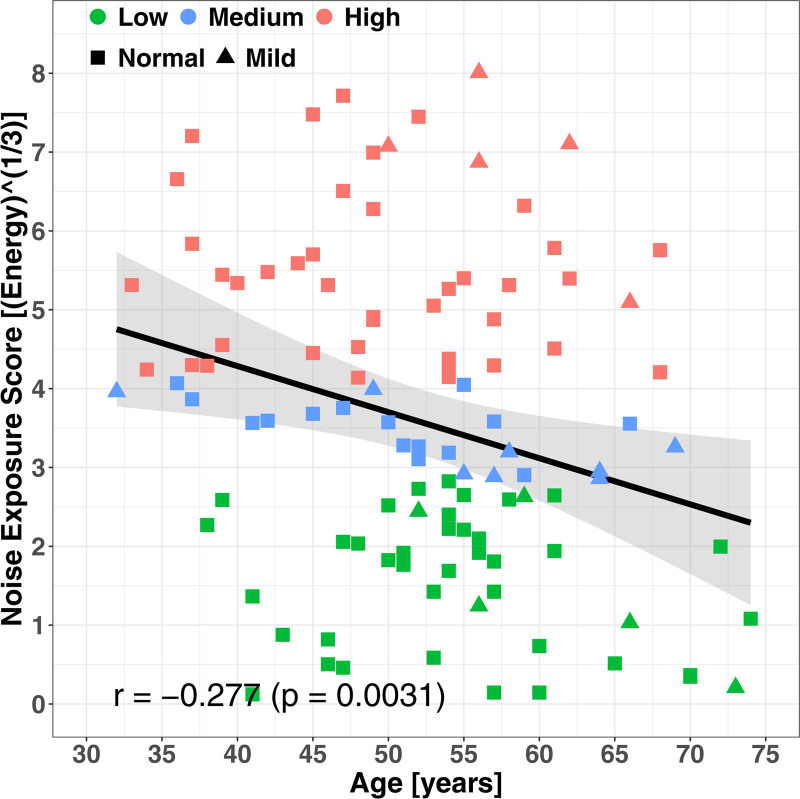
The distribution of noise exposures as a function of age, stratified by 2° of hearing (normal or mild loss) and 3° of noise exposure (low, medium, and high).

Figure 2 shows the distribution of (cube-root) cumulative NESI100 scores, as a function of age in years. The data points are shape-coded (square or triangle) according to the mean audiometric threshold as shown in the top panel of Figure 1, and color-coded according to the degree of noise exposure (green, red, or blue), as shown in the middle panel of Figure 1.

The overall range of exposure scores was between 0.12 and 8.01 [Energy^(1/3)^]. The subranges of scores were from 0.12 to 2.82 for the low group (mean = 1.56, SD = 0.87); from 2.86 to 4.07 for the medium group (mean = 3.44, SD = 0.40); from 4.14 to 8.01 for the high group (mean = 5.52, SD = 1.12). The data show a modest negative Pearson correlation of cumulative noise exposure with age (*r*(110) = –0.277, *p* = 0.0031). This finding will be discussed later.

No significant difference was observed in the NESI100 scores either between males (mean = 3.56, SD = 1.88) and females (mean = 3.58, SD = 2.07), *t*(90.22) = −0.05, *p* = 0.96, or between Normal hearing (mean = 3.54, SD = 1.95) and Mild hearing loss (mean = 3.76, SD = 2.23), *t*(22.26) = −0.403, *p* = 0.69.

### Distribution of Absolute and TEN Thresholds

**Fig. 3.**
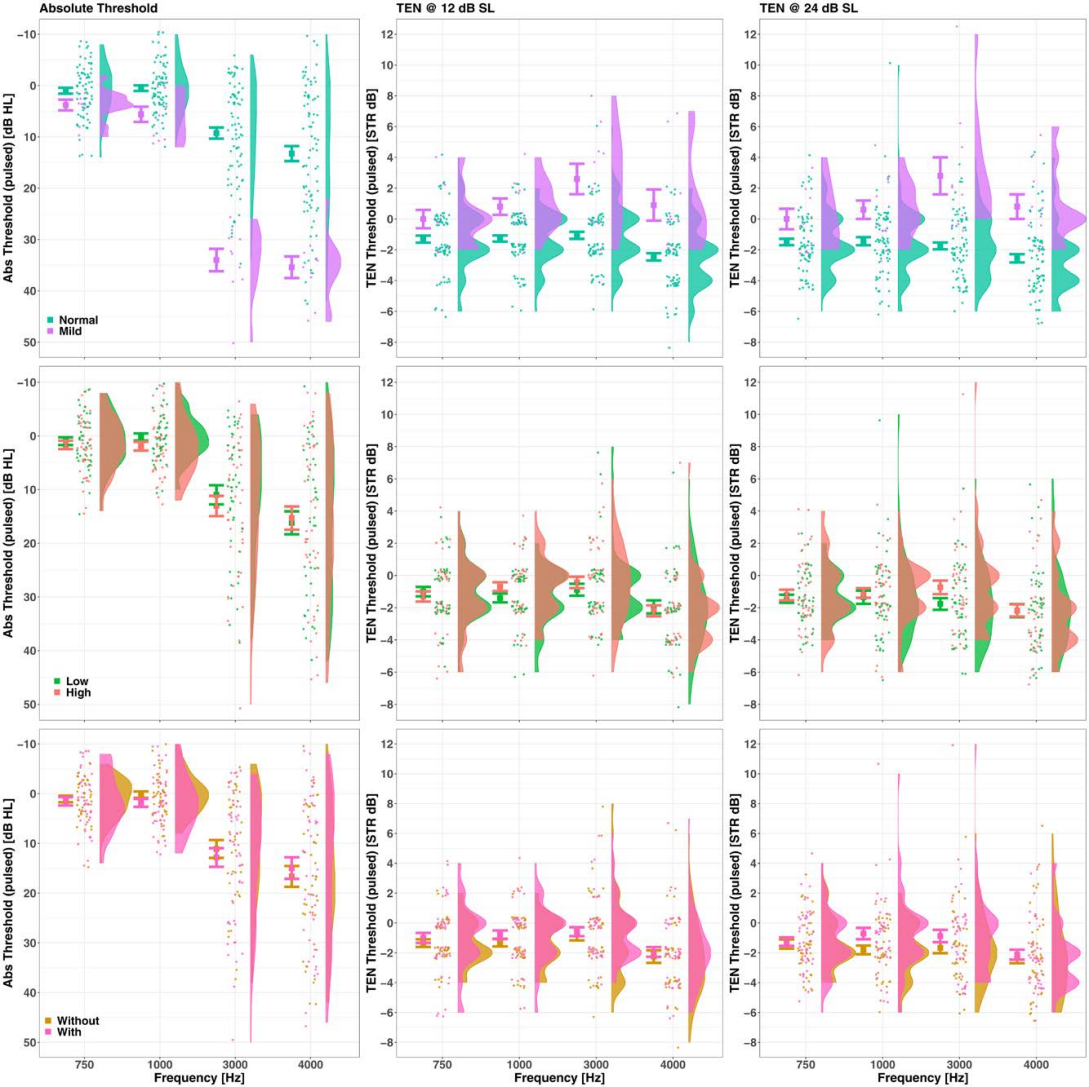
The distribution of absolute thresholds (first column) and TEN STRs (second two columns, separated by testing level) as a function of frequency, subgrouped by hearing category (top row), noise exposure (middle row), and music experience (bottom row). See text for further details.

The first column of panels in Figure 3 shows the distribution of absolute thresholds, while the next two columns show the TEN thresholds (expressed as STR), according to the groupings generated for Figure 1.

The top row shows data as stratified by degree of hearing impairment (normal/mild). The middle row shows data as stratified by degree of lifetime noise exposure, but discarding the data from the “Medium” group for clarity. The bottom row shows the data stratified by degree of music experience (Without/With). The left-hand column shows the data for the absolute threshold, the middle column for the STR at 12 dB SL, and the right-hand column the data for the STR at 24 dB SL.

The range of absolute thresholds, as measured by the pulsed tones in the unmasked portion of the TEN test, were –8 to +14 dB HL at 0.75 kHz, –10 to +12 dB HL at 1 kHz, –6 to +50 dB HL at 3 kHz, and –10 to +50 dB HL at 4 kHz. From these ranges, one can deduce that the range of noise density levels in the TEN did not exceed 40 dB HL/ERBn at the lower two frequencies; therefore, there was no level-dependent broadening of the auditory filter for these two frequencies. Although the TEN noise density varied considerably more at 3 and 4 kHz, it was still mostly below the fixed 70-dB HL/ERBn used by Ridley et al. (2018).

The overall range of the STRs was –8 to 12 dB, quantized in steps of 2 dB, and with a grand mean of –1.25 dB. Only for two participants was the TEN threshold measured at 12 dB STR, which is above the 10 dB criterion for diagnosing a dead region at a specific frequency (Moore et al. 2004). Both of these 12 dB-STR points were measured at stimulus parameters of 3 kHz and at 24 dB SL. A further two participants had STRs of 10 dB, measured at 1 and 4 kHz and at 24 dB SL. Given that the pulsed presentation was likely to improve detectability by about 2 to 3 dB, then it is reasonable to lower the criterion for diagnosis of a dead region from that of “exceeding 10 dB” to “exceeding 8 dB”. Even with such an adjustment, the incidence of a possible dead region at any frequency or level in this population was less than 0.5 %.

The plotting in the lower two rows of Figure 1, by either NESI or music experience, shows a large degree of overlap between groups.

### Analysis of Absolute Threshold Data

There were positive correlations between Age and Absolute Threshold, otherwise described in the literature as presbyacusis, for 0.75 kHz, Spearman ρ = 0.257, *p* = 0.006; for 1 kHz, ρ = 0.239, *p* = 0.011; for 3 kHz, ρ = 0.452, *p* ≤ 0.001 and for 4 kHz, ρ = 0.505, *p* ≤ 0.001. For all four frequencies, n = 112.

The absolute threshold data were best explained by a linear mixed model (Akaike Information Criterion = 2501.1, conditional R^2^ = 0.62, marginal R^2^ = 0.49) with fixed effects of Frequency [F(3, 261) = 89.78, *p* < 0.01], Hearing group [F(1, 87) = 51.44, *p* < 0.01], and their interaction [F(3, 261) = 20.97, *p* < 0.01]; the participants were entered as random intercepts. The biggest differences in absolute threshold between the Hearing groups were at 3 and 4 kHz. This result was trivial due to the criteria used for the allocation to Hearing group. Of more interest is that the absolute thresholds were similar between the groups when grouped by either noise exposure or music experience; this indicates no effect of these two factors on absolute thresholds.

### Analysis of TEN Data

There were no significant correlations between STR and Age except at 24 dB SL at 3 kHz and only when considering data with Absolute Threshold ≤ 15 dB HL (ρ = –0.273, *p* = 0.019, n = 74). The effect of controlling individually for absolute threshold, NESI100, and music experience, produced two further significant correlations, again at 24 dB SL, and 3 kHz. STR correlated with Age, when controlling for Absolute Threshold, for both the full (ρ = –0.258, *p* = 0.006, n = 112) and restricted (≤15 dB HL, n = 74) range of absolute threshold (ρ < –0.349, *p =* 0.003).

Table 1 details the significant correlations between STR and Absolute Threshold at the two test levels and four different test frequencies. For these correlations, the Medium noise-exposure group was reincluded in the data set. A general picture emerged that controlling for any of the factors NESI, Age, or music experience did not greatly affect the correlations; hence Table 1 lists only the non-controlled correlations. Simultaneous control for all three factors will be described later.

**TABLE 1.** Spearman correlations, ρ, of STRs at individual test frequencies as a function of absolute threshold (all measures obtained by use of pulsed tones)

dB SL	Frequency (kHz)	n	ρ	*p*	s
12	0.75	111	0.354	0.000	‡
	1	112	0.281	0.003	†
	3	112	0.406	0.000	‡
	4	111	0.347	0.000	‡
	3 ≤ 15 dBHL	74	0.258	0.027	*
	4 ≤ 15 dBHL	73	0.120	0.311	
24	0.75	112	0.289	0.002	†
	1	112	0.308	0.001	‡
	3	112	0.488	0.000	‡
	4	112	0.567	0.000	‡
	3 ≤ 15 dBHL	74	0.158	0.180	
	4 ≤ 15 dBHL	74	0.411	0.000	‡

Correlations were calculated either with no partialling, or partialling by NESI, age, or music experience. Since the partialling only slightly modified the significance, these variations are not reported. Each row contains the number of data points, “n,” the correlation “ρ” and the probability, *p*.

Apart from the correlations across all absolute thresholds at 3 and 4 kHz, the correlations for a data subset where only thresholds ≤ 15 dB HL are included, are shown with labels “3 ≤ 15 dB HL” and “4 ≤ 15 dB HL”.

* *p* < 0.05.

† *p* < 0.01.

‡ *p* < 0.001.

NESI, Noise Exposure Structured Interview.

The lower two lines of the two halves of Table 1 include two additional sets of correlations between STR and absolute threshold, but confining the absolute threshold at 3 and 4 kHz to be in the range of normal hearing (≤15 dB HL), which already applies to the data at 0.75 and 1 kHz. Two of the four correlations achieved significance (*p* < 0.05), 12 dB SL at 3 kHz and 24 dB SL at 4 kHz. This extends the findings of Ridley et al. (2018) who only reported such a significant correlation at 1 kHz.

**Fig. 4.**
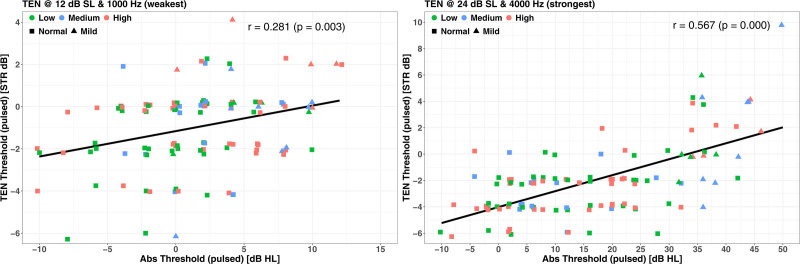
Example scatterplots for the relation between STR and absolute threshold: the weakest, STR at 12 dB SL with a test frequency of 1 kHz is shown in the left panel, while the strongest, STR at 24 dB SL with a test frequency of 4 kHz is shown in the right panel. Data points are shape and color coded as per Fig. 2, and repeated in the figure legend.

Figure 4 shows two correlation plots from which the statistics of Table 1 were compiled, ranging from the statistically weakest effect (STR at a TEN level of 12 dB SL with a 1 kHz test frequency, left-hand panel) to the statistically strongest (STR at a TEN level of 24 dB SL with a 4 kHz test frequency, right-hand panel). Note the change in both ordinate and abscissa scales between the two plots.

Comparing the data between the low and high noise-exposure groups, the same structure of linear mixed model was used as for the absolute threshold data to best explain the STR data (Akaike Information Criterion = 2960.79, conditional R^2^ = 0.61, marginal R^2^ = 0.19), using fixed effects of frequency [F(3, 260.27) = 6.279, *p* < 0.01], and hearing group [F(1, 86.89) = 42.83, *p* < 0.01], and their interaction [F(3, 260.27) = 5.31, *p* < 0.01]; with by-participant adjustments to the intercept and by-participant adjustments (i.e., random slope) to frequency. Homogeneity of variance for the participants over frequency was assumed. The STR threshold increased significantly at 3 kHz relative to the other frequencies. The mild HL group had significantly higher STRs than normal-hearing groups at 3 and 4 kHz. There were no effects of noise exposure or music experience.

### Primary Hypothesis: Elevation of STR Due to Noise Exposure in General

Our primary hypothesis, outlined in the Introduction, was that, independent of absolute threshold, the STR should be correlated with measures of noise exposure. There was no correlation found between STR and the general noise exposure metric, NESI80 (*p* > 0.05, n ≥ 111).

### Secondary Hypothesis: A Stronger Link Between STR and NESI100

Our secondary hypothesis, also outlined in the Introduction, was that the STR should be more strongly correlated with measures of noise exposure that are based on very high SPL exposures, >100 dBA, and that the correlation should be more visible in the 3 to 6 kHz region. In line with the results from previous work (Stone et al. 2008; Stone & Moore 2014), this prediction of correlation of STR with SL should be most observable at the lower testing level, of 12 dB SL.

Eight multiple regression models were run (two presentation levels × four frequencies). Consequently a Bonferroni-adjusted significance level of 0.00625 (i.e., 0.05/8) was used. The dependent variable was the STR for each combination of presentation level and frequency. The predictors were absolute threshold at the same frequency, age, NESI100, music experience, and the interaction term between NESI and age since these two were significantly correlated (Fig. 2 earlier). All predictors were standardized.

While age and absolute threshold were correlated to each other, the limited, or lack of, covarying in their correlations with STR justified their inclusion here, which was verified by the variance inflation factor lying between 1 and 1.4 (see Table 3; Howell 2012; Fox 2015). Age has been implicated in neuronal degradations observed in the human auditory periphery (Viana et al. 2015), and variation in absolute threshold will change the absolute values of testing levels, leading to covariation of the STR (Hansen et al. 2017).

Of the eight models, five reached statistical significance. The pattern of these is shown in Table 2. Of the five models achieving significance, age was only a significant predictor in two which were as follows:

**TABLE 2.** Multiple regression modeling of the STRs as a function of the predictors AbsThr, age, NESI, MExp and the interaction term, age × NESI

Dependent Variable	R^2^	Adjusted R^2^	F(df)	*p*	Sig	Durbin-Watson”s D
STR @ 12 dB SL 0.75 kHz	0.145	0.104	3.566 (5,105)	0.005	*	2.14
STR @ 12 dB SL 1 kHz	0.130	0.089	3.173 (5,106)	0.010		2.01
STR @ 12 dB SL 3 kHz	0.306	0.273	9.357 (5,106)	0.000	*	2.03
STR @ 12 dB SL 4 kHz	0.163	0.124	4.100 (5,105)	0.002	*	2.06
STR @ 24 dB SL, 0.75 kHz	0.113	0.071	2.701 (5,106)	0.024		1.89
STR @ 24 dB SL 1 kHz	0.103	0.060	2.422 (5,106)	0.040		2.12
STR @ 24 dB SL 3 kHz	0.503	0.479	21.429 (5,106)	0.000	*	1.78
STR @ 24 dB SL 4 kHz	0.428	0.401	15.873 (5,106)	0.000	*	1.67

“*p*” denotes the probability.

*“ Sig” column denotes a significant result.

AbsThr, absolute threshold; MExp, music experience; NESI, Noise Exposure Structured Interview; STR, signal-to-TEN ratio.

(1) STR @ 12 dB SL & 3000 Hz, and
(2) STR @ 24 dB SL & 3000 Hz.

NESI100 and music experience were not significant predictors in any of the eight models. Table 3 shows a summary of the coefficients from these models but only for the significant predictors of absolute threshold and age.

Overall, the models follow the pattern of the correlations earlier: at most frequencies, the STR is highly correlated with absolute threshold, but not with NESI100, and not with music experience. Only at 3 kHz do we see age as a factor alongside absolute threshold, but its relationship is *negative:* STR is modeled as improving with age, an unlikely result unless some other, unmeasured, factor, such as lifestyle, is adding heterogeneity to the participant pool. Unlike with the correlations performed using the full span of absolute thresholds in the data set (Table 1), we do not see a relationship between STR and absolute threshold at all frequencies and both testing levels.

**TABLE 3.** Table of regression coefficients derived from the multiple regression modeling of the STRs as a function of the predictors AbsThr, age, NESI100, music experience, and the interaction term, age × NESI100

Data to be modeled	Predictor	Standardized beta	CI	SE	*t*	*p*	Sig	VIF
STR @ 12 dB SL0.75 Hz	(Intercept)	–0.029	–0.215, 0.156	0.094	–0.313	0.755		
	AbsThr	0.367	0.183, 0.552	0.093	3.944	<0.001	‡	1.1
	Age	0.024	–0.168, 0.217	0.097	0.249	0.804		1.2
STR @ 12 dB SL3 kHz	(Intercept)	–0.001	–0.167, 0.165	0.084	–0.012	0.991		
	AbsThr	0.592	0.412, 0.771	0.091	6.535	<0.001	‡	1.3
	Age	–0.207	–0.393, –0.021	0.094	–2.202	0.030	*	1.3
STR @ 12 dB SL4 kHz	(Intercept)	–0.008	–0.191, 0.176	0.093	–0.082	0.935		
	AbsThr	0.397	0.194, 0.6	0.102	3.882	<0.001	‡	1.3
	Age	–0.006	–0.218, 0.206	0.107	–0.055	0.956		1.4
STR @ 24 dB SL3 kHz	(Intercept)	–0.030	–0.171, 0.11	0.071	–0.431	0.668		
	AbsThr	0.746	0.594, 0.898	0.077	9.727	<0.001	‡	1.3
	Age	–0.316	–0.474, –0.158	0.080	–3.972	<0.001	‡	1.3
STR @ 24 dB SL4 kHz	(Intercept)	–0.032	–0.182, 0.118	0.076	–0.423	0.673		
	AbsThr	0.699	0.53, 0.867	0.085	8.236	<0.001	‡	1.3
	Age	–0.139	–0.314, 0.036	0.088	–1.570	0.119		1.4

The only significant relationships depended on AbsThr and age, hence only these are detailed. The number of stars in the column “Sig” denotes the probability range of a significant effect, as detailed in the caption to Table 1.

AbsThr, absolute threshold; CI, confidence intervals; STR, signal-to-TEN ratio; VIF, variance inflation factor.

### Analysis of TFS-AF

The Pearson correlation of TFS-AF thresholds as a function of NESI was not significant (r(82) = 0.110, *p* = 0.319). Since Age was significantly correlated with both NESI scores and TFS-AF thresholds (for this latter, r(82) = –0.316, *p* = 0.003), after controlling for Age, the correlation between TFS-AF thresholds and NESI scores was insignificant: r(84) = 0.021, *p* = 0.850. This test therefore found no evidence of putative noise-induced damage to the IHC-pathway outside of the classic 2 to 6 kHz region where noise-related damage is first observed in the human audiogram.

The Spearman correlation of TFS-AF thresholds as a function of STR at 1 kHz (the frequency closest to the bulk of the TFS-AF thresholds) was not significant either at 12 dB SL (ρ = 0.045, *p* = 0.681, n = 84) or at 24 dB SL (ρ = 0.109, *p* = 0.322, n = 84).

A multiple linear regression model was run on TFS-AF using PTA (derived as the average of the audiometric thresholds at 1 and 2 kHz), Age, NESI and music experience as well as the interaction term between NESI and age as predictors. The model was significant [R^2^ = 0.207, adjusted R^2^ = 0.156, F(5, 78) = 4.079, *p* = 0.002]. The significant predictors of TFS-AF were age (standardized beta = –0.289, *p* = 0.011) and music experience (standardized beta = 0.316, *p* = 0.003). We have replicated the link of TFS-AF thresholds with age that has been shown before (Füllgrabe et al. 2018), but not with low-frequency hearing loss, possibly because of our inclusion criteria, which would limit the range of losses included.

## DISCUSSION

### Measuring the Degradation of the Auditory System

The motivation for this work was that a quickly administered clinical test, the TEN test, could provide more information about the frequency-specific patency of the hearing system than just that provided by the audiogram. A noise-exposure measure was also included to address the modern concerns that noise, specifically recreational, rather than industrial, in origin (in high- and middle-income countries), is the main driver of “modern” noise-induced hearing loss (NIHL), especially in young adults (Smith et al. 2000).

The data presented here do not support the hypothesis of a linkage between elevation of the STR in the 3 to 4 kHz region in the TEN test and high-level (>100 dBA) noise exposure. The data do show another association that expands our understanding of the gradual decline of the human auditory system over the course of the lifespan. Our data provide a strong link between a form of hearing deficit, the elevation of STR, and absolute thresholds, even when absolute thresholds at a wide range of individual frequencies were clinically “normal.” This is in agreement with the data shown by Ridley et al. (2018) in a much more limited design (a single test frequency of 1 kHz, fewer participants (N = 20), a single, much higher testing level (70 dB HL/ERBn), and with no control for age, noise exposure or music experience). A similar observation in animals, of normal absolute threshold but abnormal auditory performance (Lobarinas et al. 2017), specifically identified by a site of lesion (Kujawa & Liberman 2009), spawned the loosely related field of cochlear synaptopathy research.

We do not attribute the observed elevation of STR within the range of “normal” absolute thresholds to the effect of altered auditory filter shape. Neural tuning curves in animals have been successfully modeled as a parallel combination of a low-sensitivity, wide-bandwidth linear filter (the “tail filter”) and a high-sensitivity, sharply tuned filter (the “tip filter”) whose output is nonlinear with level, but whose sharp tuning is invariant with level (Goldstein 1990). The combined output of these two filters then give rise to the observed effects of broadening with level. Therefore, TEN testing at low SLs, as well as combined with low levels of audiometric loss, should primarily reflect neural output from the tip filter alone.

### Consistent Observation of Elevation of the STR Predicted by the Absolute Threshold

The slope of the elevation of the STR as a function of absolute threshold was 1/5 at 1 kHz and 1/7 at 4 kHz, units of dB/dB HL as plotted in Figure 4 of Ridley et al. (2018). Examples of the corresponding slopes reported here were 1/10 at 0.75 kHz and 1/7 at 3 kHz (Fig. 4). It should be remembered that the two sets of slopes were measured at very different testing levels, with a very different hypothesis driving each experiment. The intriguing aspect of these slopes, even significant where absolute thresholds at these frequencies were “normal” in both studies, suggests that human hearing function degrades from an early age, measures of its function involves the interaction of multiple cochlear structures (Schuknecht & Gacek 1993; Viana et al. 2015), and that truly “normal” hearing on an audiogram (i.e., undamaged) is more of a line than a band.

Similar observations have been made with measures of Distortion Product OAEs: strength of the emission has been shown to correlate with pure-tone absolute threshold, even for values of absolute threshold below 20 dB HL at the test frequency (Dorn et al. 1998). However, in that study, these relationships disappeared when a stricter definition of absolute threshold being “normal” at all audiometric frequencies was assumed.

### Correlations of Cumulative Noise Exposure with Age

Figure 2 showed a significant negative correlation of cumulative noise exposure with age. The data of Smith et al. (2000) would lead one to expect a significant positive correlation due to the reported increased opportunities for noise exposure from the early 1990s. With a younger cohort, our Manchester-based group has previously shown positive correlations with age (Pearson *r* = 0.52, *p* < 0.01) among 126 young participants aged 18–36 years, barely overlapping with ours (Prendergast et al. 2017b). Expanding on the age range, Prendergast et al. (2019) recruited 33 extra older people, up to age 59 years, with a mean of 44.8 years. The full set still showed a Spearman correlation of exposure with age ρ = 0.5 (*p* < 1^–10^), but the correlation among the older participants was insignificant (ρ = 0.24, *p* = 0.17).

One explanation is that younger and older populations are not following the same life course (aside from possible differences in recall between the age groups): the younger population are acquiring a cumulative exposure faster than their antecedents, a manifestation of the more recent increased access to high-sound levels (Smith et al. 2000). This effect will gradually ripple through the population in these cumulative measures to older participants, but over the next 20 to 30 years. This observation relies on the accuracy of historical recall, which is commonly questioned in the literature (Ridley et al. 2018; Section 1.3.10 in Bramhall et al. 2019).

### Accuracy of Cumulative Noise Exposure Estimates Exceeding 100 dBA

The lack of effect of the NESI exposure tool to reveal a link of cumulative noise exposure with absolute threshold is notable, especially since accumulated dose forms the basis of predicted damage in medico-legal cases (Coles et al. 2000). There was very little difference in the results from the statistical analyses whether we used the NESI score for exposures exceeding either 100 dBA or exceeding 80 dBA. Estimates of cumulative exposure are most sensitive to the estimate of sound level since this is expressed in logarithmic units, while exposure time (and accumulation) is in linear units. Due to previous work, we confined our NESI estimates to exposures exceeding 100 dBA. However, as mentioned earlier, debate surrounds the accuracy of estimates of historic noise exposure. Ferguson et al. (2019) reported that use of the speech effort scale to estimate noise levels (as used in the NESI) typically had a mean difference of approximately 3 dB, for exposures levels between 87 and 93 dBA. However, at levels of 99 dBA, this mean difference nearly doubled, to just under 6 dB. This implies that estimates of exposures to levels in the high 90-dB range and above, may be prone to large errors. Some of this error will have been truncated by our preference for the one-third power transform in statistical analyses (and our attempts during transcribing to ensure that noise estimates were credible). However, the Ferguson et al. work may part-explain the difficulty here in obtaining measurable difference in effects between the two exposure limits we used for NESI calculations.

### The Quest for Psychophysical Evidence of Subclinical Noise-induced Damage

A recent review of the evidence for noise-induced synaptopathy in humans suggests that one reason for the many contradictory findings may be the variability in the populations studied (Section 1.3.6 in Bramhall et al. 2019). Toppila et al. (2001) tested over 700 participants to model the degree of NIHL due to industrial exposures, ranging from 70 to 125 dBA, with a mode of 103 dBA. Although they found that chronological age was a strong predictor, it was confounded by the effects of some of the biological factors that they also measured because these confounders had accumulated effects with age. Their modeling therefore placed little weight on elapsed age per se, unless dealing with older workers, but much more weight was given to other lifestyle factors such as cholesterol level, blood pressure, and the use of clinical pharmaceuticals whose effects accumulate over time. Their conclusion was that, as the number of confounders increased (and they listed other studies that had used biological measures other than theirs), the relation between and age and NIHL reduced.

This observation by Toppila et al. (2001) could explain why our low-SL testing of older participants failed to find any similar effects of perceptual deficits at low SLs due to noise exposure, despite effects being reported in other low-SL experiments but where much younger participants had been used (Stone et al. 2008; Vinay & Moore 2010; Stone & Moore 2014). The negative correlation of NESI with Age, despite NESI being a cumulative measure, as well as the linear mixed modeling showing a negative dependence of Age on STR (at 3 kHz and both testing levels) implies that the participants' lifestyles were not homogeneous over time, again in line with the suggestions from Toppila et al. (2001). An explanation for the negative NESI relationship with Age is that older participants may have poorer recall of events more remote in time. The regression slope (beta in Table 2) of STR with Age at 3 kHz and 24 dB SL was –0.316 (confidence interval between –0.474 and –0.158). For the 40-year age span of our data, this would translate into an underlying STR range of 12 dB. This figure spans almost the complete range of STR one would expect to measure in the “sick” region of the TEN test, and of similar size to dilute the effect of any other factor. If these negative relationships are true, then it indicates a potential confound.

Our data appear to go a step beyond the observations of Toppila et al. in that, in the correlations, there were minimal effects of noise exposure, music experience, and age (beyond that on absolute threshold and STR at 3 kHz). This would support the postulate of Bramhall et al. (2019) that (usually unintentional) bias in participant selection can completely obliterate any measurable effects of other oto-toxic processes. Unless the effects are gross, these quests for evidence of noise-induced damage can be “mission (near) impossible” (Bramhall et al. 2019).

## CONCLUSIONS

A group of 112 participants ranging in age from 32 to 74 years were selected from a larger pool by screening for clinically normal hearing at 0.75 and 1 kHz, and questioned about lifetime noise exposure at high-sound levels by use of the NESI. They performed the TEN test at four frequencies, and at two levels, 12 and 24 dB, above absolute threshold. The selection by normal hearing at low frequencies, as well as lack of a conductive component to their hearing thresholds was intended to select for people with postnatally acquired hearing damage, if any.

Correlational analyses showed the following:

(1) A strong contribution of aging to the elevation of absolute threshold, the classical definition of presbyacusis.
(2) No link between degree of noise exposure and elevation of absolute threshold.
(3) Across a wide range of center frequencies, the elevation of the TEN threshold into the “sick” region between “pass” and “fail” was (a) almost entirely driven by the elevation in absolute threshold, and (b) occurred even when the absolute threshold was within a “normal” range, even when drawn more stringently (≤15 dB HL) than the clinically conventional ≤20 dB HL. Although some elevation of TEN threshold has previously been reported at high testing levels, such high testing levels were rarely used here due to the selection criteria and thresholds encountered.

We conclude that an elevation of the TEN threshold less than the “fail” criterion:

(1) appears to reflect a general degradation of multiple cochlear mechanisms, primarily related to OHC dysfunction because of its strong dependence on elevation of absolute threshold, rather than with dysfunction in the IHC-pathway.
(2) occurs at a rate of 1 dB for every 7 dB of absolute threshold elevation above 0 dB HL.
(3) is not sensitive enough to indicate putative effects of noise damage, and therefore should not be used as such in a clinical setting other than as a pass/fail decision tool.

These data, derived from a clinical rather than a laboratory tool, do not support the previous findings in much younger cohorts by Stone et al. (2008), Vinay & Moore (2010), and Stone & Moore (2014) concerning evidence of noise-induced damage being measurable at low SLs. In addition, this clinical tool does not give evidence to the hypothesis that the pattern of cochlear damage changes depending on the profile of the noise exposure, such as exposures exceeding 100 dBA. We suggest that the older and wider age range employed here introduced a heterogeneity into our participant pool that obscured the observation of any effects. The possible clinical use of the TFS-AF test to reveal noise-induced IHC-related dysfunction was also not supported.

The data presented add further support to Smith et al. (2000) who reported that high-level noise exposures have become more common in the general population over the past 30 years.

## ACKNOWLEDGMENTS

This work was funded by the UK Medical Research Council (M.A.S., W.B., C.J.P.), reference MR/M023486/1 and by the NIHR Manchester Biomedical Research Centre (M.A.S., E.P., M.L., H.W., C.J.P.).

## Supplementary Material


